# The rhizosphere bacterial community contributes to the nutritional competitive advantage of weedy rice over cultivated rice in paddy soil

**DOI:** 10.1186/s12866-022-02648-1

**Published:** 2022-09-30

**Authors:** Yue Wu, Jian Sun, Pengcheng Yu, Weiliang Zhang, Youze Lin, Dianrong Ma

**Affiliations:** Rice Research Institute, Shenyang Agricultural University, Shenyang, China

**Keywords:** Weedy rice, Rhizosphere bacterial, Nutritional competition, 16S ribosomal RNA

## Abstract

**Background:**

Weedy rice competes for nutrients and living space with cultivated rice, which results in serious reductions in rice production. The rhizosphere bacterial community plays an important role in nutrient competition between species. It is therefore important to clarify the differences in the diversities of the inter rhizosphere bacterial community between cultivated rice and weedy rice. The differences in compositions and co-occurrence networks of the rhizosphere bacterial community of cultivated rice and weedy rice are largely unknown and thus the aim of our study.

**Results:**

In our study, the different rhizosphere bacterial community structures in weedy rice (AW), cultivated rice (AY) and cultivated rice surrounded by weedy rice (WY) were determined based on 16S rRNA gene sequencing. The majority of the WY rhizosphere was enriched with unique types of microorganisms belonging to *Burkholderia.* The rhizosphere bacterial community showed differences in relative abundance among the three groups. Network analysis revealed a more complex co-occurrence network structure in the rhizosphere bacterial community of AW than in those of AY and WY due to a higher degree of Microbacteriaceae and Micrococcaceae in the network. Both network analysis and functional predictions reveal that weedy rice contamination dramatically impacts the iron respiration of the rhizosphere bacterial community of cultivated rice.

**Conclusions:**

Our study shows that there are many differences in the rhizosphere bacterial community of weedy rice and cultivated rice. When cultivated rice was disturbed by weedy rice, the rhizosphere bacterial community and co-occurrence network also changed. The above differences tend to lead to a nutritional competitive advantage for weedy rice in paddy soils.

**Supplementary Information:**

The online version contains supplementary material available at 10.1186/s12866-022-02648-1.

## Introduction

Weedy rice (*Oryza sativa* f. *spontanea*) poses a major threat to paddy fields worldwide since it has the typical properties of invasive weeds, such as strong reproductive ability, invasiveness, and high phenotypic plasticity, in addition to a high capacity to compete for resources [1, 2]. The decreased rice yields caused by weedy rice indicate that it is able to compete with cultivated rice for nutrients with complex and diverse competition mechanisms.

Multiple plant traits are associated with the richness of microorganisms in the soil environment [3]. A number of microorganisms are involved in the transformation of soil nutrients, including the cycling of nitrogen and phosphorus [4, 5]. The root systems of different plants selectively recruit rhizosphere microorganisms from the soil, which means that in different plant species, species-specific microbial communities are present. The individual gene differences among plant hosts may have a large impact on the rhizosphere microbiome [6]. Factors that affect the microbial composition of plant roots also include the soil types, geographical locations, growth and development status of plant hosts, microbial interactions and others [7–9]. For example, the rhizosphere pH and root Cd content have significant associations with the rhizosphere bacterial assembly of rice in Cd-contaminated paddy fields [10]. Different planting locations and different genotypes significantly affected the community structure of the rice rhizosphere microbiome. A study showed that N inputs decreased ammonia-oxidizing bacteria (AOB) diversity but increased the dominance of the genus *Nitrosospira* within the AOB community in a Tibetan alpine meadow [11]. Another study showed that significant differences in rhizosphere microbiome diversity exist between the *indica* and *japonica* rice subspecies, which directly affects the efficiency of nitrogen fertilizer use [12].

The rhizosphere microbiome can also have an impact on the growth, development and health of plant hosts. Beneficial microbial communities contribute to plant growth, directly or indirectly, by increasing the nutrient uptake of plants, as well as by protecting against pathogens [13–15]. For example, AMF can participate in nutrient exchange in the plant–soil system and play an important role in promoting the degradation of soil organic matter or enhancing its nitrogen fixation capacity. Many bacteria, including *Bacillus*, *Enterobacter*, *Citrobacter*, *Burkholderia*, *Panobacter*, *nitrogen-fixing bacteria* and *Pseudomonas,* have the ability to dissolve phosphate that plants can later uptake [16]. The microbial symbiosis mechanism between plant roots is very important, and direct mechanisms often use microbial traits to directly promote plant growth [17]. Hence, the crop rhizosphere microbiome can influence the nutritional competitiveness, yield and quality of the crop in many ways [18, 19].

Weedy rice and cultivated rice show significant differences in seedling growth status, tiller capacity, plant height, plant shape and seed set per plant. Weedy rice has a higher competitive ability regarding nutrients than cultivated rice [20]. However, whether this difference in competitive ability is related to the rhizosphere microbiomes of both species has not yet been reported, and the effect of the association of weedy rice on the rhizosphere bacterial communities of cultivated rice is unclear. Therefore, in this study, the rhizosphere bacterial communities of weedy rice and cultivated rice were studied by using 16S rRNA gene sequencing technology. Through multiple analyses, we compared the differences between the rhizosphere bacterial community of weedy rice and cultivated rice and changes in the rhizosphere bacterial community of cultivated rice when weedy rice was present. Our results provide some references for an in-depth understanding of the composition between the rhizosphere bacterial communities of weedy rice and cultivated rice in northern China and to investigate the nutrient competition mechanism between the two from the perspective of rhizosphere bacterial communities.

## Results

### Diversities of the rhizosphere bacterial communities of different samples

High-throughput sequencing resulted in a total of 208,165 optimized sequences and 78,404,538 bases with an average sequence length of 376 bp from nine samples. The sequencing results were extracted from the optimized sequences using UPARSE 7.0 to decrease the number of redundant calculations in the middle of the analysis, and single sequences without repeats were removed, which resulted in a total of 1155 OTUs. Dilution curves (Fig. S1) and rank-abundance curves (Fig. S2) were constructed to better represent the richness of the flora in the samples between treatments and whether the amounts of sequencing data were reasonable. The dilution curves flattened out as the number of amplifications increased, which indicated that the sequencing data were reasonable and that the sample sizes were adequate for this sequencing. The rapid, steep declines in the rank-abundance curves indicate high proportions of the dominant colonies in the samples and low levels of diversity in the rhizosphere microbial community compositions.

The species richness and diversity in the samples were compared by using alpha diversity analysis. Table 1 includes a series of statistical analysis indices that were used to estimate the species abundances and diversities in the environmental communities, and the results of the between-group difference test demonstrate that the host growth environment and genotype did not significantly affect the species richness of the rhizosphere microorganisms (*P* > 0.05).

**Table 1:** Primer sequences used to amplify the 16S rRNA gene

Primer name	Primer sequence	Length	Sequencing platforms	Amplification region
799F	5´-AACMGGATTAGATACCCKG-3´	593 bp	Miseq PE300	Second round of amplification of the V5-V7 region
1392R	5´-ACGGGCGGTGTGTRC-3´			
799F	5´-AACMGGATTAGATACCCKG-3´	394 bp		
1193R	5´-ACGTCATCCCCACCTTCC-3´			

### Changes in the rhizosphere bacterial community composition caused by genotype and habitat

**Fig. 1:**
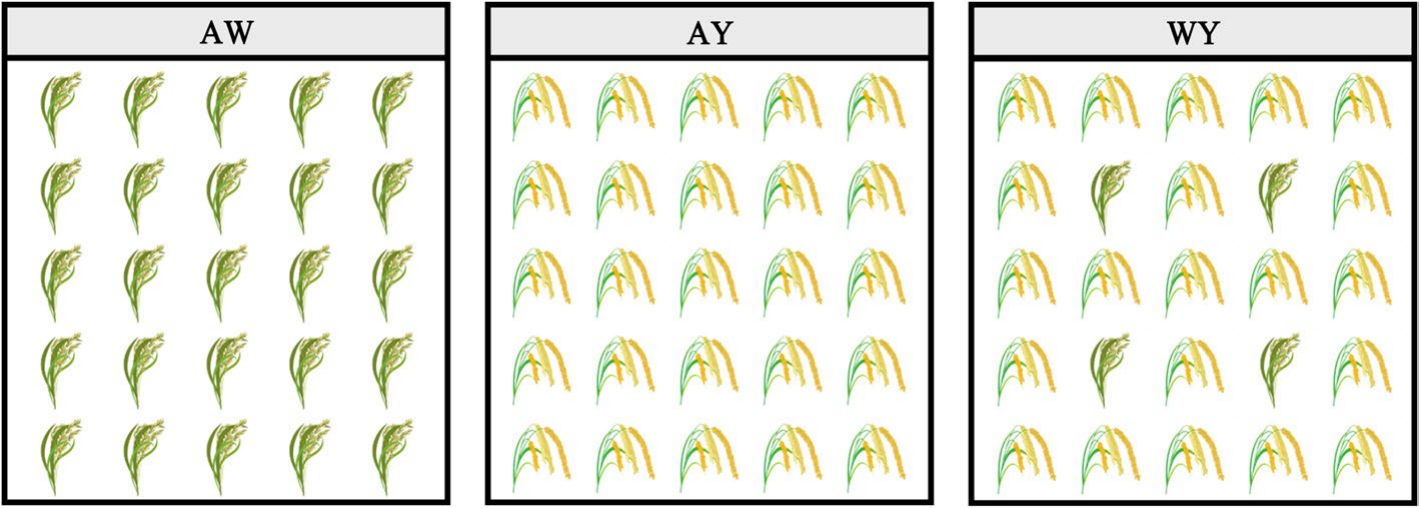
Diagram of the experimental design for rice field trials. AW: weedy rice square; AY: cultivated rice square; and WY: cultivated rice square with weedy rice. Harvested samples for each variety were surrounded by protected plants that separated the different varieties

PLS-DA (Fig. 1) showed that the weedy rice group (AW) and cultivated rice group (AY) were clearly separated at COMP2, which indicated that there were significant differences in the rhizosphere bacterial community compositions of the weedy and cultivated rice at the OTU level. Furthermore, the cultivated rice surrounded by concomitant weedy rice (WY) and cultivated rice alone (AY), they were clearly separated at COMP1 and were located in the same position on the first axis, which suggested that the presence of concomitant weedy rice around cultivated rice had a significant effect on the rhizosphere bacterial community composition of the cultivated rice rhizosphere, which implied that the host growth environment was another important factor that may have contributed to the differences among the samples.

**Fig. 2:**
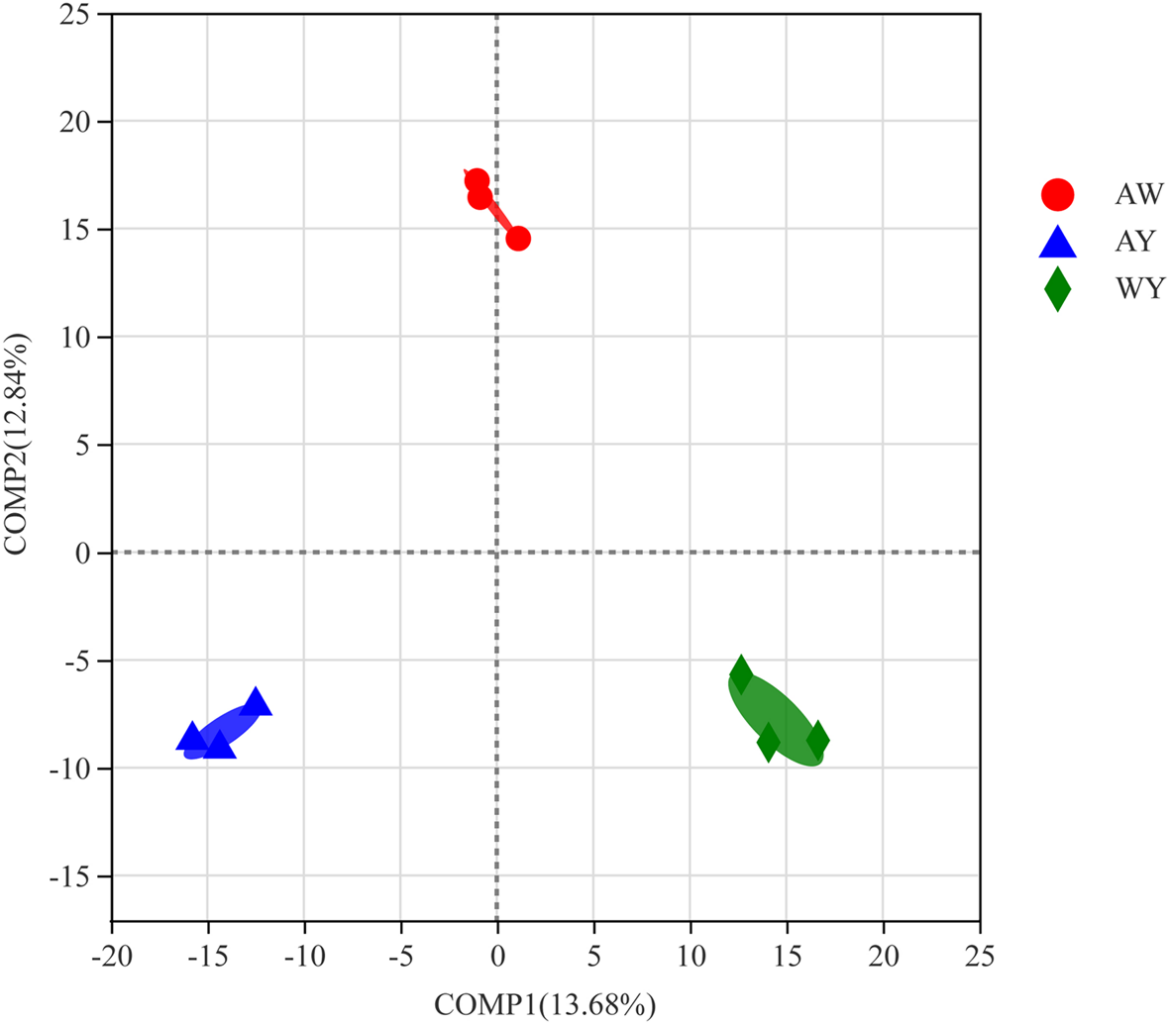
PLS-DA analysis at different sample OTU levels. Different coloured or shaped dots represent groups of samples in different environments or conditions. The AY and WY groups separated at COMP1; AW is separated from AY and WY at COMP2

A Venn diagram analysis (Fig. 2) revealed that 662 OTUs in the rhizosphere bacterial communities overlapped between the three groups at the OTU level, with the highest number of unique OTU types (75) appearing when cultivated rice was surrounded by associated weedy rice, and the differentially enriched bacteria belonged mainly to the phylum level classifications of Abditibacteria, Berkelbacteria, AKAU4049 and vadinHA49. The next highest number was found for the weedy rice group (68), whichhad five different phyla. The cultivated rice group has the fewest unique types (61), all of which belong to Gracilibacteria at the phylum level (Table S1).

### Abundance differences in the rhizosphere bacterial community

**Fig. 3:**
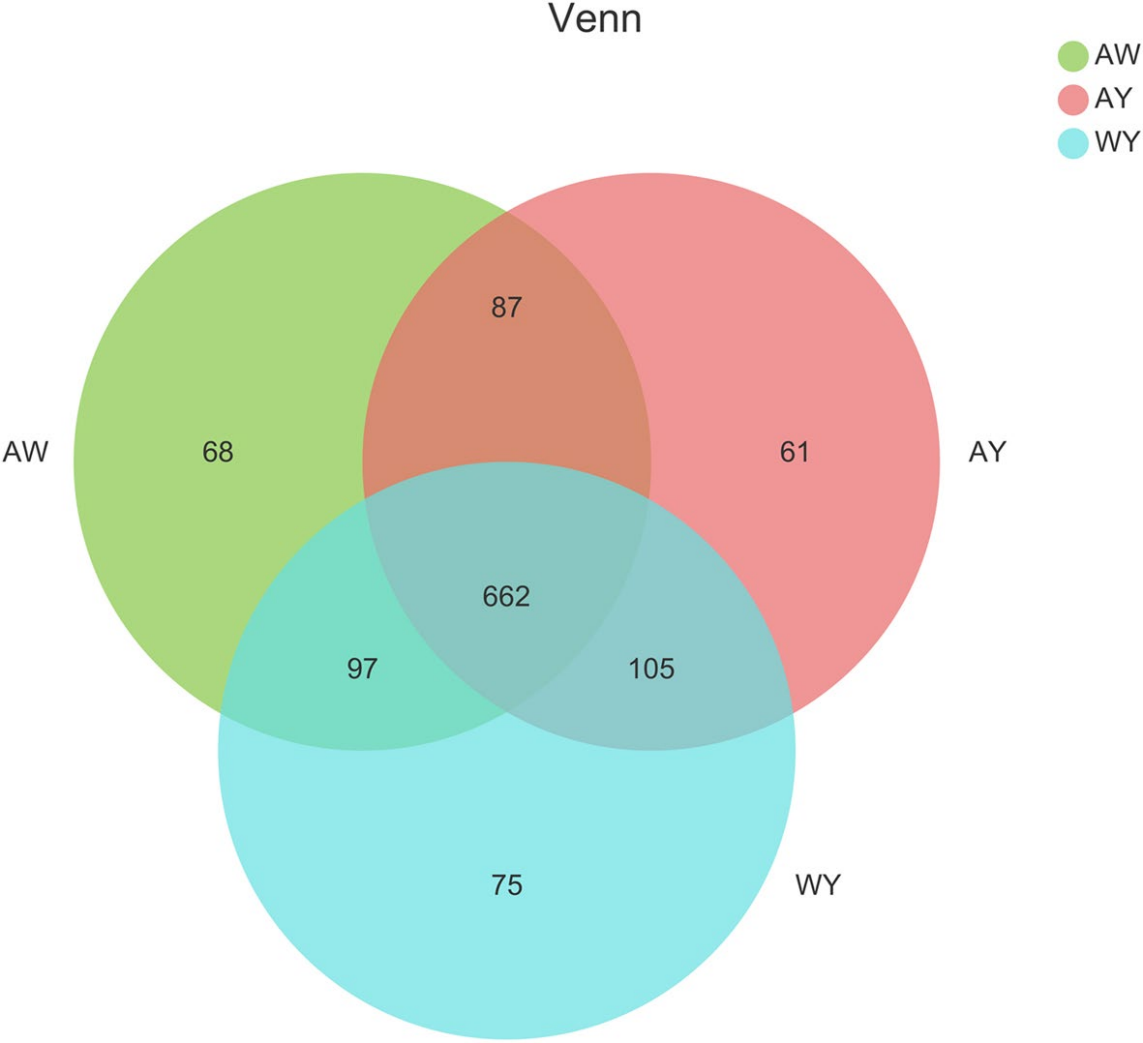
Differences in the compositions of the rhizosphere bacterial community at the OTU level for different samples. The numbers in the overlapping part represent the number of OTUs common to the three groups, and the numbers in the nonoverlapping part represent the number of OTUs specific to the corresponding group

The comparison of the differences in relative abundance between the rhizosphere bacterial communities of weedy rice and cultivated rice at different taxonomic levels (Fig. 3a) revealed that at the family level, weedy rice and cultivated rice differed in their abundances of Nakamurellaceae, Smithellaceae, Koribacteraceae and KD3-93. The abundances in weedy rice were significantly higher for Smithellaceae and KD3-93 than those in cultivated rice and were significantly lower for Nakamurellaceae and Koribacteraceae (Welch's t test, *P < 0.05; **P < 0.01).

When cultivated rice was surrounded by weedy rice, the cultivated rice rhizosphere bacterial community showed significant changes at the family level (Fig. 3b), with Xanthobacteraceae, Pedosphaeraceae, Leptospiraceae, PHOS-HE36, norank_o __Gaiellales, and norank_o__WCHB1-4 being significantly less abundant, while the abundances of Micromonosporaceae, Exiguobacteraceae, Geodermatophilaceae, and Sulfuricellaceae were significantly lower.

In addition, after comparing the abundance of the rhizosphere bacterial community in weedy rice and that in cultivated rice surrounded by weedy rice, it was found that the abundance of Planococcaceae, Geothermobacteraceae, Mycobacteriaceae and KD3-93 was significantly higher in weedy rice rhizosphere than in cultivated rice surrounded by weedy rice rhizosphere (Fig. 3c).

### Differences in rhizosphere bacterial community co-occurrence networks

**Fig. 4:**
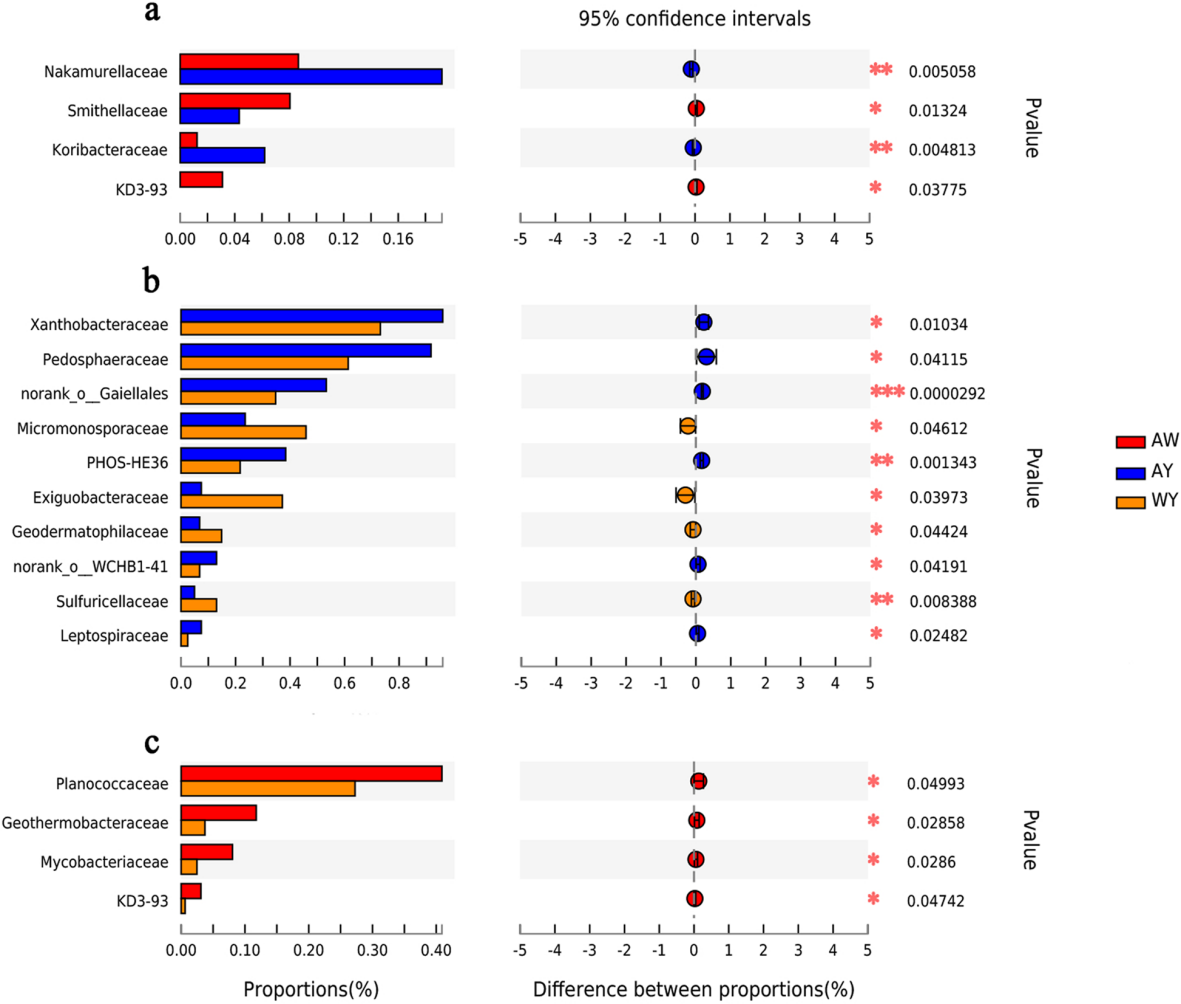
Species showing the abundance differences in the rhizosphere bacterial communities at the family level. **a** Weedy rice vs. cultivated rice. **b** Cultivated rice vs. cultivated rice surrounded by weedy rice. **c** Weedy rice vs. cultivated rice in association with weedy rice. Bar plot displaying the differences in the family levels of bacterial communities among the three groups. Asterisks represent the significance of correlation (*, *P* < 0.05, **, *P* < 0.01, ***, *P* < 0.001, Welch’s t test)

Microbial co-occurrence networks at the family level for weedy rice and cultivated rice were constructed and analysed to determine the correlations between the bacteria in different networks and the differences in the connectivity of shared bacteria. Each node in the network represents a family, and the node size indicates the average relative abundance of that family in each set of samples, with the interactions between two families connected by line segments. Degree indicates the number of nodes directly connected to the node in the network, with a higher degree indicating a higher importance of the node in the overall network. For example, Sphingomonadacea had 26 and 25 degrees in the AW and WY groups, respectively, while it had only 12 degrees in the AY group (Table S2). In total, the AW group co-occurrence network contained the highest number of degrees (838); the WY group had 834 degrees, while the AY group had only 768 degrees (Table S2). The co-occurrence network diagrams for the three groups differed markedly (Fig. 4), with weedy rice and cultivated rice associated with weedy rice being more complex than cultivated rice alone in terms of the densities of the connecting lines. The interrelationships among microorganisms also differed in the co-occurrence networks of the different groups. For example, Sphingomonadaceae was negatively correlated with Desulfocapsaceae and Syntrophobacteraceae in the AW group, while it was positively correlated in the other two groups. In addition, in the AW group, Sphingomonadaceae was negatively associated with a total of eight families, while this number reached 19 and 14 in the AY and WY groups, respectively.

The differences between groups at the family level also differed, with different genotypes and environments affecting the microbial co-occurrence networks, as shown in Table 2. Among them, Xanthobacteraceae and Micromonosporaceae were present in the weedy rice rhizosphere co-occurrence network but not in the cultivated rice rhizosphere co-occurrence network; however, both microorganisms reappeared when the cultivated rice was accompanied by weedy rice, which demonstrated that the presence of weedy rice affected the cultivated rice rhizosphere bacterial community co-occurrence network.

**Table 2:** Alpha Diversity Indices of different samples

	Community richness			Community evenness	
Sample	sobs	ace	chao	shannon	simpson
AW	618.00 ± 55.027	904.69 ± 85.891	860.81 ± 88.239	4.10 ± 0.551	0.15 ± 0.077
AY	624.00 ± 30.643	878.89 ± 51.228	867.94 ± 40.887	4.34 ± 0.316	0.11 ± 0.041
WY	643.33 ± 53.144	969.65 ± 97.461	965.85 ± 94.579	4.19 ± 0.441	0.14 ± 0.062
P (AW-AY)	1	0.663	1	1	0.663
Q (AW-AY)	1	1	1	1	1
P (AY-WY)	0.663	0.383	0.19	0.663	0.663
Q (AY-WY)	0.663	0.663	0.571	0.663	0.663
P (AW-WY)	0.663	0.663	0.19	1	1
Q (AW-WY)	0.994	0.994	0.571	1	1

Note: Data in the table are the mean  ±  standard deviation (Mean  ±  Sd), P is the probability of false-positive values, and Q is the FDR value

**Fig. 5:**
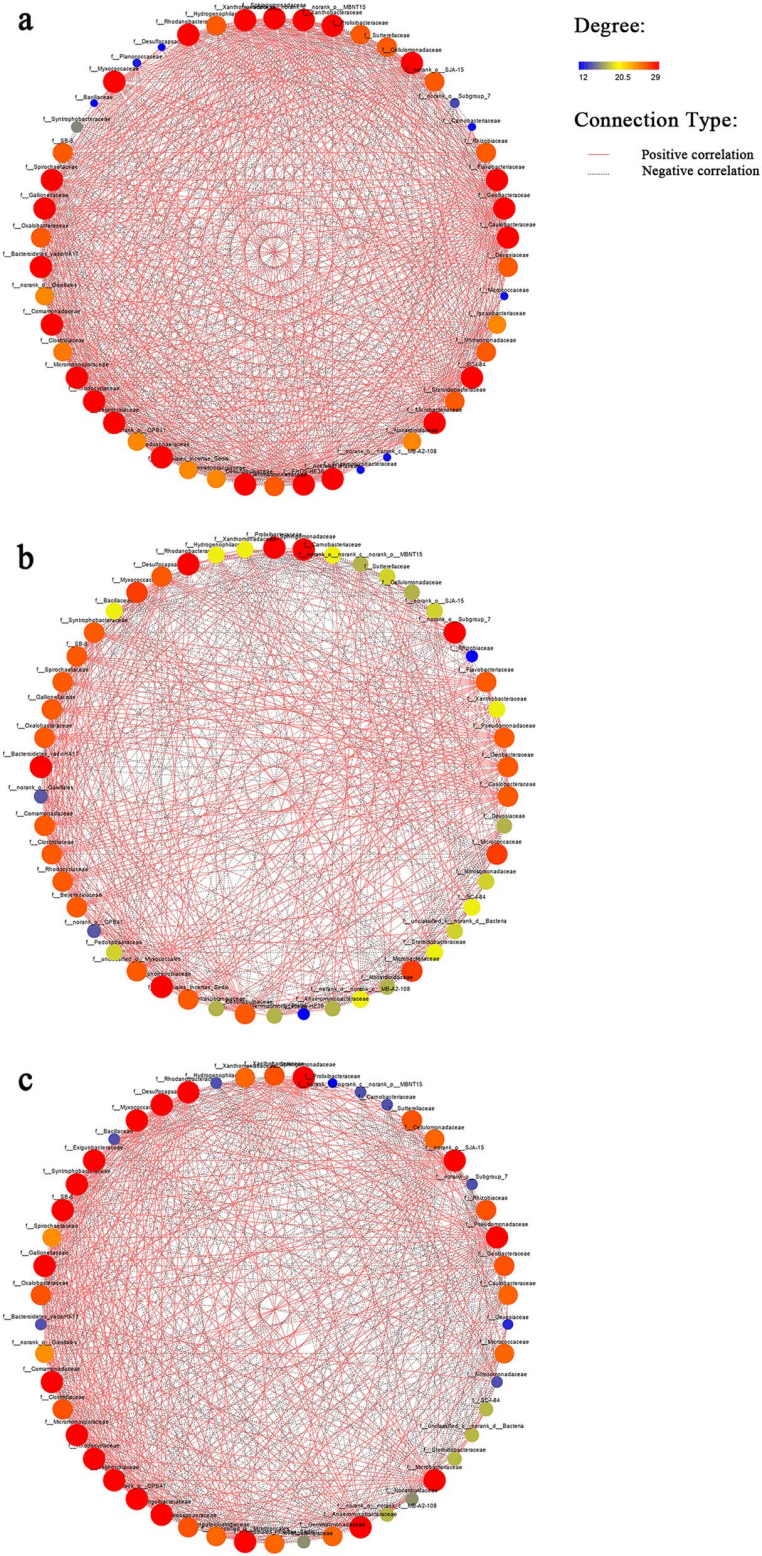
The co-occurrence networks of the rhizosphere bacterial community of (**a**) weedy rice (AW), (**b**) cultivated rice (AY) and (**c**) cultivated rice surrounded by weedy rice (WY) were constructed. The size of each node is proportional to the number of connections. Each node is marked at the family level. A connection represents a strong (|r|> 0.5) and significant (*P* < 0.05) correlation (Spearman's rank correlation coefficient)

To quantify the differences in co-occurrence networks among groups, this study compared the shared bacteria in the different networks and their connectivities. The heatmap of the node degree of the shared bacteria (Fig. 5) shows that there were significant differences among some of the shared bacteria in the weedy rice and cultivated rice networks. For example, for Micrococcaceae, Microbacteriaceae, Pedosphaeraceae, Myxococcaceae and PHOS-HE36*,* the node degree in the weedy rice network was 26, while it was significantly lower in the cultivated rice network map. In contrast, the bacteria with higher node degrees in the cultivated rice network included Steroidobacteraceae, Desulfocapsaceae, Syntrophobacteraceae, and Rhizobiales Incertae Sedis, which was higher than the 17 node degrees for weedy rice.

The heatmap of the node degree of the common bacteria in the cultivated rice rhizospherein different survival environments showed that some of the bacteria also differed significantly between the two networks (Fig. 5). For example, for Nitrosomonadaceae, Microbacteriaceae, Myxococcaceae, norank_o__OPB41, and norank_o__SJA-15 in the cultivated rice rhizosphere network with weedy rice competition, the node degree was 25. The differences compared to cultivated rice were over 18 degrees. The above result implies that significant changes in the co-occurrence network were found when cultivated rice was surrounded by weedy rice. This suggests that differences in host survival environments strongly influence the structure of rhizosphere bacterial co-occurrence networks.

### Functional prediction of the rhizosphere bacterial community using FAPROTAX analysis

**Fig. 6:**
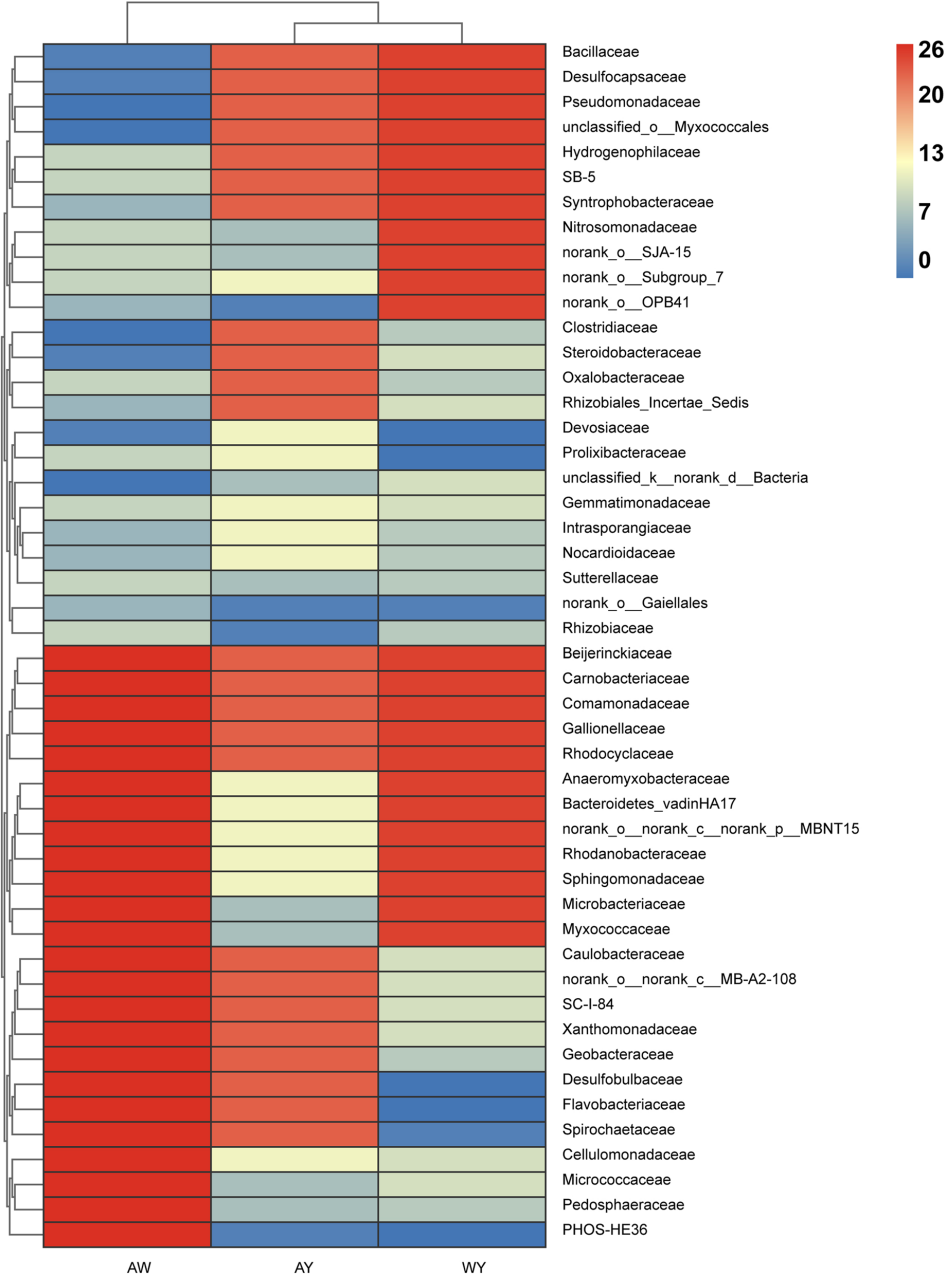
Heatmap of the node degree between the three groups of co-occurrence networks of shared bacteria at the family level. There were 48 bacteria in the three networks, and different colours indicate the degree of association of each bacterial as a node with other bacteria in the network

FAPROTAX analysis was utilized to evaluate the difference in the functional rhizosphere bacterial community within the three groups (AW, AY and WY). A total of 43 functional annotation results were detected by FAPROTAX, and the abundance of OTUs enriched in different groups is shown in Table S3. The domain grouping of functions was linked to biogeochemical cycling, such as C (34.88%), N (23.26%), S (16.28%) and Fe (4.65%). Figure 6 shows the top 20 functional abundances in the three groups. We found that 15 functionally enriched OTUs were relatively lower in the WY group than in the AY group, four were higher and one were at a similar level. Moreover, a relatively higher abundance of OTUs was detected in the WY group than in the AY group. This suggests that the accompaniment of weedy rice generally inhibits the functional activity of the rhizosphere bacterial community in cultivated rice. We found that the abundance of bacterial groups associated with iron-respiration was lowest in the WY group, with an abundance 41% lower than that in the AY group. Additionally, 4 functions associated with N cycling were slightly higher in the WY group than in the AY group.

## Discussion

### The differences in the rhizosphere bacterial community of weedy rice, cultivated rice and cultivated rice surrounded by weedy rice

Weedy rice occurs to varying degrees throughout the world [1, 21–23]. In recent years, with the spread of direct seeding and no-till cultivation methods, weedy rice has become increasingly prevalent [24–26], and causes serious impacts on rice production worldwide [27]. The competitive advantage of weedy rice over cultivated rice is manifested in several ways. However, the literature literature has mostly addressed the competition between weedy rice and cultivated rice in the aboveground part, such as plant height, leaf width and length, and tiller capacity [28], with relatively little competition in the belowground part, and few studies have been reported on competition from the perspective of the rhizosphere bacterial community [29].

The compositions of plant rhizosphere microbial communities are closely related to the host plants. The host genotype is an important factor that influences the composition of rhizosphere microbes, which has been demonstrated in a variety of important crops, such as maize [30] and rice [31]. It was found that *indica* and *japonica* rice recruit different rhizosphere microbiomes in the field. *Indica* rice has a higher level of microbial diversity than *japonica* rice, including more genera related to nitrogen metabolism [31]. In this research, cultivated rice and weedy rice were found to recruit different rhizosphere bacterial communities in the field. Furthermore, when cultivated rice was surrounded by associated weedy rice, there were also significant differences found in the rhizosphere bacterial community (Fig. 1), suggesting that the host survival environment is another influential factor affecting differences in the composition of the rhizosphere bacterial community. Microflora abundance tests at the family level indicated that both genotype and habitat significantly influenced the bacterial enrichment levels (Fig. 3). Interestingly, the effect of changing growth environments on microbiota abundances appears to be more pronounced, as reflected by the larger number of species with significantly different family-level abundances across growth environments compared to those across different genotypes. A further comparison of the rhizosphere bacterial communities in weedy rice and when cultivated rice was surrounded by associated weedy rice showed that each of the bacteria that appeared to differ in abundance had a higher abundance in the rhizosphere of weedy rice (Fig. 3c). In particular, Geothermobacteraceae is a kind of thermophilic bacterium in the family Geobacteraceae, which is contains the main Fe (III)-reducing microorganisms [32]. The higher abundance of Geobacteriaceae in the AW group implies greater iron-reducing capacity. Rhizobacterial communities are a complex mix of factors that have very large impacts on plant nutrition and subsequent plant growth [33]. Therefore, it may be a new research direction to determine the mechanism for the competitive advantage of weedy rice over cultivated rice from the perspective of the rhizosphere bacterial community.

### Weedy rice significantly affects the rhizosphere bacterial community of cultivated rice and subsequently its nutrient acquisition and growth

The rhizosphere bacterial community is involved in the regulation of various life activities, such as plant growth, development and stress resistance [34, 35]. For plants, the bacterial community can extend its metabolic capabilities, including nutrient acquisition, immune regulation and resistance to biotic or abiotic stresses [36]. For example, a study found large differences in the composition of inter- and intra-rhizosphere bacteria of millet, with the abundance of bacterial taxa associated with nitrogen fixation being significantly higher in wild millet roots than in domesticated modern millet varieties [37]. Compared to cultivated rice, the inter-rhizosphere zone of wild rice is richer in bacterial communities such as slow-growing rhizobia, which facilitate plant acquisition of N and P, and *Streptomyces*, which enhance plant resistance [38]. *Xanthomonas* has been found to be associated with basic diseases on many crops, such as potatoes and bananas [39]. In our study, Xanthobacteraceae appeared in the rhizosphere bacterial community of cultivated rice when weedy rice was present in the surrounding area (Table 2), which possibly affected the healthy growth of cultivated rice. In addition, when cultivated rice is surrounded by weedy rice, the majority of its rhizosphere is enriched with unique types of bacteria belonging to *Burkholderia*. It has been demonstrated that *Burkholderia* exerts a negative regulatory role in the mineralization of soil nitrogen to inorganic nitrogen, which may be a cause of impaired rice growth and development [40].

### Co-occurrence network structure may lead to a competitive advantage for weedy rice

Complex microbial-microbial interactions are an important factor that mediate changes in the dynamics of microbial community structures [41, 42]. Plant rhizosphere microbial co-occurrence networks can enable visualization of the correlations among different microorganisms, which is crucial for finding interactions among bacteria [43]. In the natural environment, the variety and number of microorganisms and the complexity of extraneous elements make it difficult to apply laboratory results. In recent years, additional research has focused on describing the co-occurrence networks between the microbial communities in natural samples [44]. The co-occurrence characteristics within microbial communities are affected by a variety of environmental factors. For example, a study on the structure of the rhizosphere flora of wild and domesticated rice showed that the co-occurrence network complexity is higher in wild rice rhizosphere fungal community than in that of domesticated rice [45]. The higher values of connectivity and nodes in the co-occurrence network make the network of the wild rice rhizosphere more complex than that of the domesticated rice rhizosphere. Consequently, this enables the rhizosphere of wild rice to be more stable in response to changes in the external environment [46]. Communities with greater commonality and more negative/positive connections, have more complex network structures. Such communities may be more resistant to environmental disturbances, resulting in greater stability [47]. In our study, by comparing the rhizosphere bacterial community co-occurrence networks of weedy rice and cultivated rice, we found that the networks of weedy rice had higher degrees and nodes, which made the network of the weedy rice rhizosphere more complex and stable than that of the cultivated rice rhizosphere. This may lead to a competitive advantage in the growth of weedy rice. In addition, interactions between some rhizosphere bacterial communities showed differences in weedy rice and cultivated rice. For example, Microbacteriaceae and Micrococcaceae, with higher degrees in the weedy rice rhizosphere bacterial community network map, were shown to be significantly and positively correlated with the rate of soil soluble organic nitrogen conversion [48]. In contrast, Oxalobacteraceae, a higher degree family in the cultivated rice rhizosphere bacterial community network map, was significantly and negatively correlated with the rate of soil soluble organic nitrogen conversion [49]. This implies that the weedy rice rhizosphere bacterial network has fewer bacterial interactions that reduce the rate of conversion of soil soluble organic nitrogen. These interactions between the rhizosphere bacterial communities may be another reason for the greater competitiveness of weedy rice. However, it is not clear how weedy rice improves plant nutrient competition, nutrient use efficiency and stress tolerance through the selective recruitment of beneficial bacterial communities. We need to continue to identify the main regulatory genes that are involved in selective bacterial recruitment and to discover the mechanisms of regulation at the community level of the bacteria that play key roles.

### Key microbial species in co-occurrence networks may lead to a competitive advantage in weedy rice

Higher microorganism abundances do not indicate key roles in co-occurrence networks. For instance, the relative abundances of most microorganisms of the important key nodes are very low, namely, less than 0.5% [50, 51]. Critical microbial species may play important roles in maintaining network stability, as their disappearance could lead to separations of modules and networks [40, 52]. This study found that when cultivated rice was surrounded by weedy rice, the cultivated rice rhizosphere bacterial co-occurrence network became more complex, and the composition of the co-occurrence network was very different. In addition, there were distinct changes in the node degrees of the cooccurring bacteria in the network, which suggested that the emergence of weedy rice had a dramatic impact on the cultivated rice rhizosphere bacterial co-occurrence network. Geobacteraceae, which have a high degree in the cultivated rice network in the absence of competition from weedy rice, are microorganisms commonly found in ferric reducing environments. Microorganisms employ a decrease in metabolic iron to convert extracellular insoluble ferric iron oxides Fe (III) to ferrous iron Fe (II). The most well-known dissimilatory Fe (III) reducers belong to the Geobacteraceae family [53]. This process is central to several other biogeochemical cycles in various anoxic environments, which, for instance, significantly influence the carbon, nitrogen, and phosphorus cycles [54]. Therefore, the diversity and abundance of Geobacteraceae communities in paddy soils could provide valuable information to improve the soil fertility and productivity of paddy soils [55]. Among the genera that were shared by both wild and cultivated rice, Geobacter had a higher abundance in the rhizospheres of wild rice accessions and was identified as one of the hub taxa in the rhizosphere bacterial communities of wild rice accessions [55, 56]. In our study, the node degrees of Geobacteraceae in the co-occurrence network of weedy rice and cultivated rice reached 26 and 23, which indicated that they play a pivotal role in the networks. However, the node degrees of Geobacteraceae in the rhizosphere bacterial co-occurrence network of cultivated rice clearly decreased when weedy rice was present, which indicated that its role in the network was weakened. This may be one of the reasons for the reduced nutrient acquisition capacity of cultivated rice and is a direction for future research.

It is well known that reproduction of weedy rice populations is the result of natural selection, while that of cultivated rice is the result of artificial selection. For this reason, we infer that the direction of the selection pressure on crops shapes the differentiated bacterial communities in the rhizosphere. In conclusion, continuing to dig deeper into rhizosphere bacterial co-occurrence network maps to identify the role of key differential bacterial groups may provide a breakthrough in further exploring how weedy rice acts on its own growth and development through the rhizosphere bacterial community and competes with cultivated rice for nutrients.

### Functional differences in the rhizosphere bacterial community of weedy rice, cultivated rice and cultivated rice surrounded by weedy rice

FAPROTAX is a manually constructed database for predicting the function of bacteria and archaea in terrestrial and marine ecosystems [57]. A number of studies have used FAPROTAX to predict and analyse the diversity and function of soil bacteria in different environments and thus to analyse some of the environmental factors that determine functional microbial communities in soils [58, 59]. Iron plays an important role in biochemistry in natural environments [60, 61]. FAPROTAX analysis shows that different groups have different abundances in Fe cycling. The WY group had the lowest abundance of iron-respiration-related functional compounds. This is consistent with our previous results on the node degree of Geobacteraceae in co-occurrence networks. The higher abundance of Geobacteriaceae in the AW group and AY group implies greater iron-respiration capacity. In the WY group, the lower abundance of Geobacteriaceae implies weaker iron-respiration. This suggests that the presence of weedy rice near cultivated rice reduces the ability of the cultivated rice root system to respire iron. Based on our results, some functional rhizosphere bacterial communities may change following variations in environmental factors, such as host genotype and growth environment. However, the FAPROTAX application is limited by the size of the database and taxonomic identification [62]. Similar to this study, a gap may exist between the soil functional microbial groups predicted by FAPROTAX and the real soil ecological function. Therefore, in the future, we need to continue to deepen our research on the contribution of the rhizosphere bacterial community to the nutritional competitive advantage of weedy rice in paddy soil.

## Conclusions

Our study shows that there are many differences in the rhizosphere bacterial communities of weedy rice and cultivated rice and that when cultivated rice is disturbed by weedy rice, the bacterial communities change, and these changes result in a nutritional competitive disadvantage for cultivated rice in paddy soils.

### Materials and methods

#### Seed disinfection, germination and transplanting

**Fig. 7:**
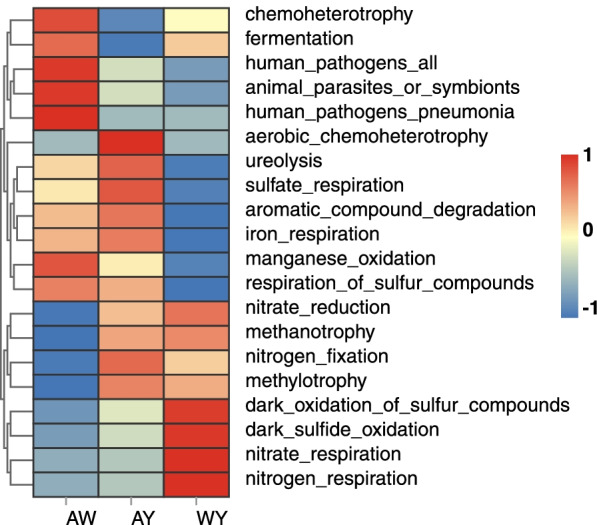
Functional Annotation of Prokaryotic Taxa (FAPROTAX) analysis was utilized to evaluate the abundance of bacteria within the three groups. The horizontal coordinate indicates the name of the function, and the vertical coordinate indicates the value of the abundance of a function for that sample, with different colours indicating different groups. **a** FAPROTAX analysis was utilized to evaluate the abundance of carbon transformation rhizosphere bacterial communities; (**b**) FAPROTAX analysis was utilized to evaluate the abundance of nitrogen transformation rhizosphere bacterial communities; and (**c**) FAPROTAX analysis was utilized to evaluate the abundance of iron and sulfur transformation rhizosphere bacterial communities

Full seeds of cultivated and weedy rice were selected, the dried seeds were discarded, the seed glumes were removed without damaging the embryo, and the seeds were placed in sterile triangular vials and sterilized using alcohol. A quantity of 70% alcohol was added, the seeds were covered with the liquid and disinfected for 30 s, the vials were shaken continuously to ensure that each seed was in full contact with the alcohol, and the alcohol was then discarded. To sterilize with NaClO, an NaClO solution with an effective chlorine concentration of 2.5% was added, the seeds were covered with the solution and sterilized for 15 min, the vials were shaken continuously during this time, and the NaClO was then discarded. The samples were washed with sterile deionized water after 3 repetitions. Sterile deionized water was added, the seeds were covered with the liquid and washed for 10 min, the vials were shaken continuously, and the sterile water was discarded. This step was repeated 3 times before inoculating the media. By using sterile forceps, the seeds were spread neatly and evenly on the surface of the MS solid medium, with the embryos placed upwards on the bottom 1/3 of the plate, andsealed with Parafilm sealing film. Sterile seedlings were obtained by culturing the seeds for 5 days by orienting the Petri dishes vertically. To prevent the residual nutrients in the medium from affecting plant growth and development after transplanting, the root residual medium needed to be removed. Sterile plant seedlings with uniform growth were selected, the sterile seedlings were pulled out of the medium using sterile forceps, and any residual medium was washed away from the roots with sterile deionized water. The sterile seedlings were transplanted to the field for culture. The sterile seedlings were transplanted to the field in squares of 5 × 5 plants, with different squares arranged as shown in Fig. 7. There were 20-cm distances between the rice plants in the squares, and the distance between squares was 30 cm. There was a total of 8 weeks of growth under normal water and fertilizer conditions. The different squares were replicated three times each.

#### Rice cultivation, sample collection and plant traits

The plants located on the borders of the plots were designated for protection and were not harvested. Root samples were taken eight weeks after seedlings were planted in the fields during the late tillering stage. Three representative individuals from each rice variety were selected from central positions. The samples were dug out with a complete soil block around them, and the rice plants were split evenly in half from the base of the stalks. Whole roots were cut from 2 to 3 tillers in the fault layer and shaken vigorously in 30 mL of sterile deionized water until the soil particles adhering to the root surface were dislodged. The roots were picked out with sterile forceps and discarded. The remaining liquid was centrifuged at 3000 rpm for 15 min. A portion of the supernatant was discarded until approximately 5 mL of soil sediment and supernatant were retained and mixed well. All samples were immediately stored at –20 C, transported to the laboratory on dry ice and stored at -80 C.

#### DNA extraction, PCR amplification and illumina sequencing

The microbial community genomic DNA was extracted from the soil samples using the DNeasy® PowerSoil® Pro Kit (QIAGEN, U.S.) according to the manufacturer’s instructions. The DNA extracts were checked on a 1% agarose gel, and the DNA concentrations and purities were determined with a NanoDrop 2000 UV–vis spectrophotometer (Thermo Scientific, Wilmington, USA).

The first round of amplification of the V5-V7 region of the 16S rRNA gene was performed using the PCR primers, 799F and 1392R [63, 64] (Table 3). PCR amplification of the 16S rRNA gene was performed as follows: initial denaturation at 95℃ for 3 min, which was followed by 27 cycles of denaturation at 95℃ for 30 s, annealing at 55℃ for 30 s and extension at 72℃ for 45 s, and a single extension at 72℃ for 10 min, with a hold at 4℃. The PCR mixtures contained 4 μL of 5 × *TransStart* FastPfu buffer, 2.5 μL of 2 mM dNTPs, 0.8 μL of forward primer (5 μM), 0.8 μL of reverse primer (5 μM), 0.4 μL of *TransStart* FastPfu DNA Polymerase, 10 ng of template DNA, and ddH_2_O to form a 20-μL volume. The PCRs were performed in triplicate. The PCR products were extracted from 2% agarose gels, purified using the AxyPrep DNA Gel Extraction Kit (Axygen Biosciences, Union City, CA, USA) according to the manufacturer’s instructions and quantified using a Quantus™ Fluorometer (Promega, USA).

**Table 3:** Different bacteria in the groups in the family-level co-occurrence network

AW vs. AY		AY vs. WY	
AW	AY	AY	WY
*Xanthobacteraceae*	*Hyphomicrobiaceae*	*Hyphomicrobiaceae*	*Xanthobacteraceae*
*Micromonosporaceae*	*Clostridiaceae*	*Flavobacteriaceae*	*Micromonosporaceae*
*Ignavibacteriaceae*	*Pseudomonadaceae*	*Devosiaceae*	*Sanguibacteraceae*
*Acetobacteraceae*	*unclassified_o__Myxococcales*	*Prolixibacteraceae*	*Hungateiclostridiaceae*
	*unclassified_k__norank_d__Bacteria*	*Desulfobulbaceae*	*Exiguobacteraceae*
		*PHOS-HE36*	

The resultant products were mixed according to the sequencing volumes required for each sample, and a second round of amplification was performed using 799F and 1193R [65] (Table 3). The PCR conditions were the same as those for the first round, with a cycle number of 13. The libraries were constructed using the NEXTFLEX® Rapid DNA-Seq Kit, and the library templates were enriched using PCR amplification. The purified amplicons were pooled in equimolar amounts and paired-end sequenced on an Illumina MiSeq PE300 platform (Illumina, San Diego, USA) according to the standard protocols used by Majorbio Bio-Pharm Technology Co. Ltd. (Shanghai, China). The data reported in this paper will be deposited in a public database before the manuscript is published.

#### Bioinformatics analysis of 16S rRNA gene profiling

The raw 16S rRNA gene sequencing reads were demultiplexed, quality-filtered with fastp version 0.20.0 [66] and merged with FLASH version 1.2.7 [67] by using the following criteria for simultaneous quality control and filtering of sequence quality. The operational taxonomic units (OTUs) with a 97% similarity cut-off [68, 69]were clustered using UPARSE version 7.1 [68], and the chimeric sequences were identified and removed. The taxonomy of each representative OTU sequence was analysed by RDP Classifier version 2.2 [70] against the 16S rRNA database SilvaRelease138 (http://www.arb-silva.de) by using a confidence threshold of 0.7. Multiple diversity index analysis was conducted based on the OTUs using Mothur software. Based on the taxonomic information, statistical analyses of the community structures could be carried out at different taxonomic levels.

## Supplementary Information

**Additional file 1. Fig.S1.**

**Additional file 2. Fig.S2.**

**Additional file 3. Table S1. **Different bacteria in the groups in the phylum level classifications.

**Additional file 4. Table S2. **Node names and their degree in co-occurrence network diagrams of different groups.

**Additional file 5**.** Table S3. **The relative abundance of functional groups in each of the stands among weedy rice (AW), cultivated rice (AY) and cultivated rice surrounded by weedy rice (WY).

## Data Availability

The 16S rRNA gene sequencing data on which the conclusions of the manuscript rely have been deposited in the National Center for Biotechnology Information (NCBI) database (accession number: PRJNA856640).
