# Supplementary figures and images for: The rhizosphere bacterial community contributes to the nutritional competitive advantage of weedy rice over cultivated rice in paddy soil
Source: BMC Microbiol. 2022 Sep 30;22:232. doi: 10.1186/s12866-022-02648-1 (PMC9523940; doi:10.1186/s12866-022-02648-1)

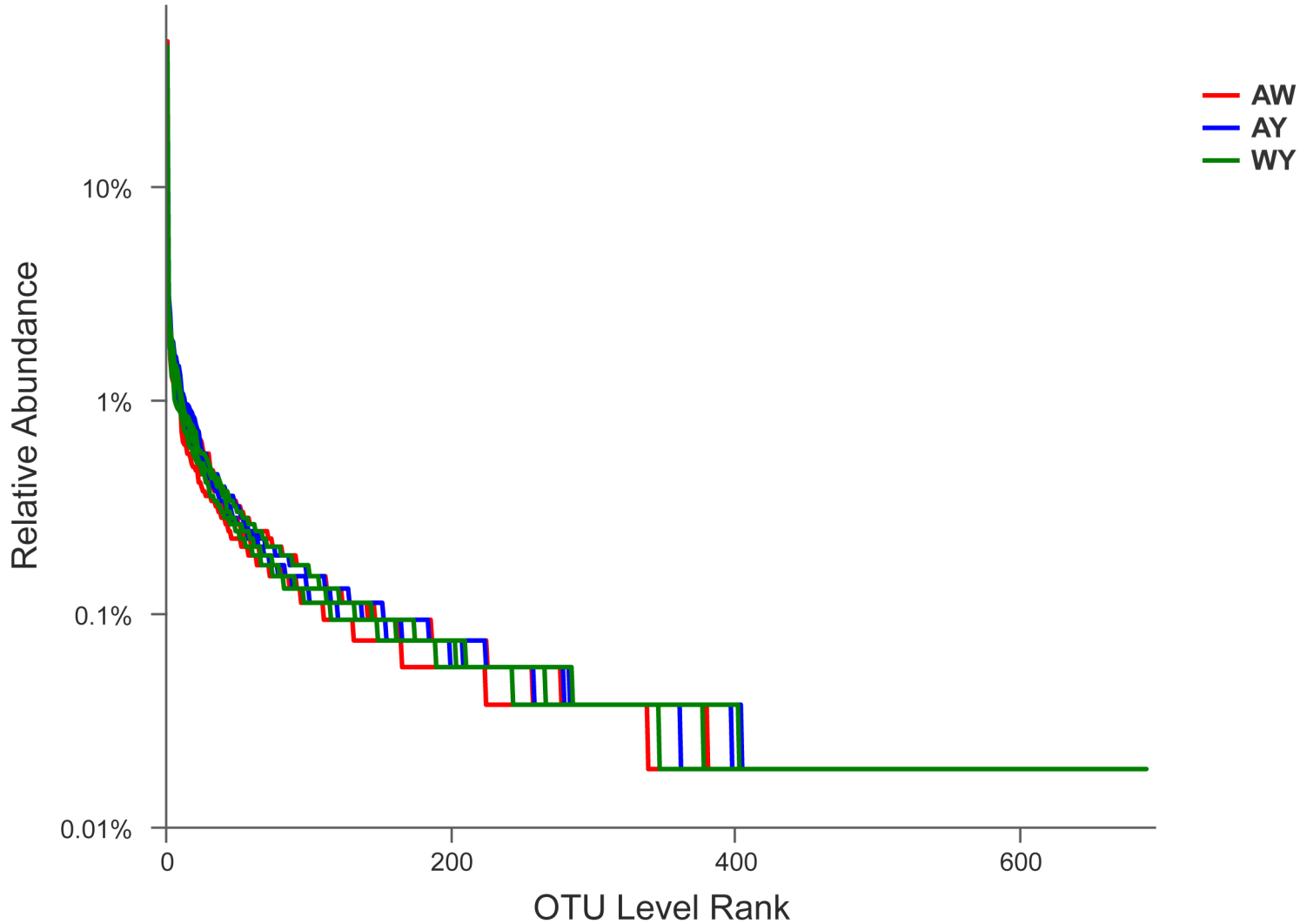

Supplement: Supplementary file 1 — Additional file 1. Fig.S1. [file 12866_2022_2648_MOESM1_ESM.pdf]

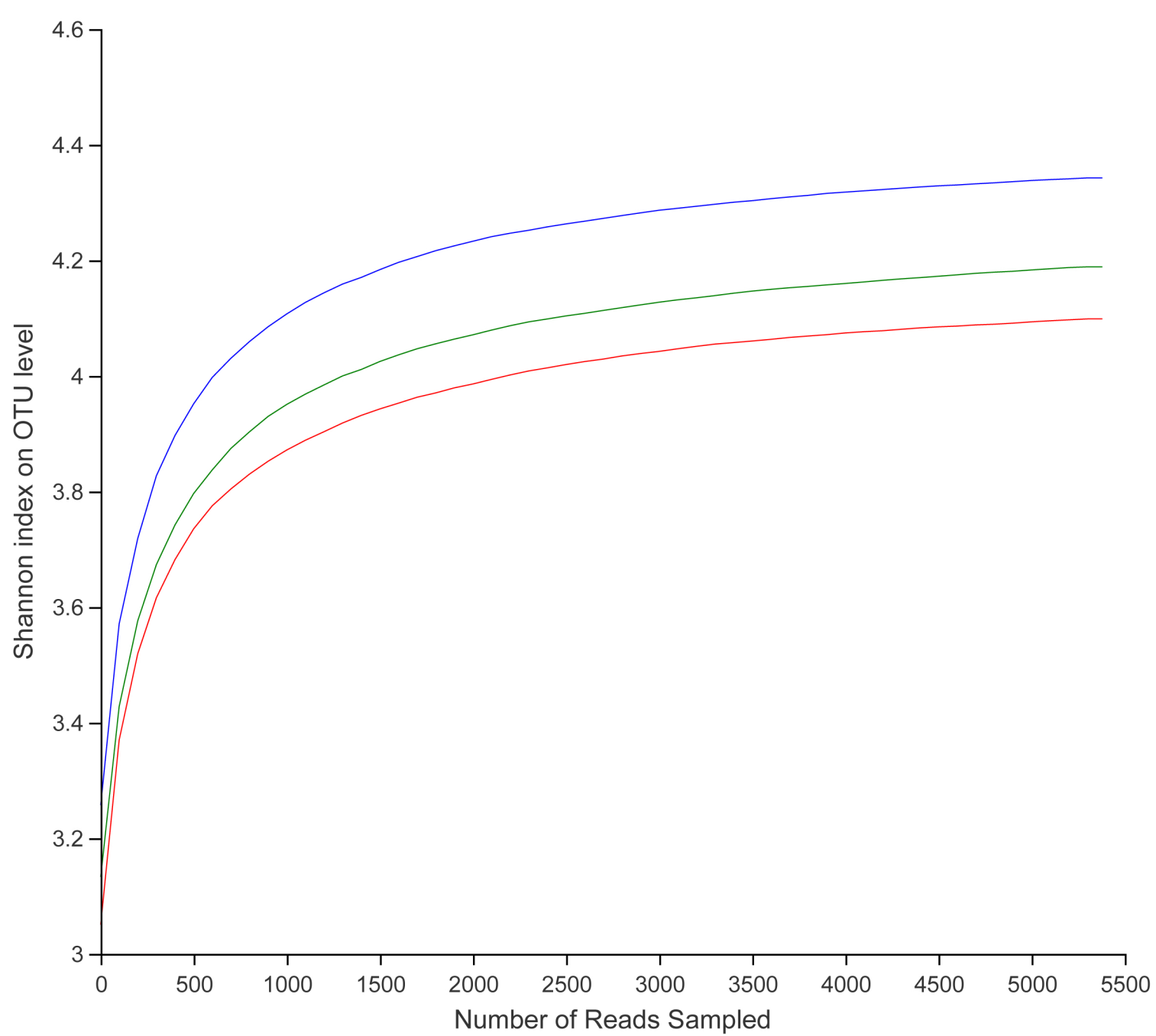

Supplement: Supplementary file 2 — Additional file 2. Fig.S2. [file 12866_2022_2648_MOESM2_ESM.pdf]
